# Family-focused contextual factors associated with lifestyle patterns in young children from two mother-offspring cohorts: GUSTO and EDEN

**DOI:** 10.1186/s12966-022-01266-4

**Published:** 2022-03-15

**Authors:** Airu Chia, Alexandra Descarpentrie, Rene N. Cheong, Jia Ying Toh, Padmapriya Natarajan, Ray Sugianto, Shirong Cai, Cécilia Saldanha-Gomes, Patricia Dargent-Molina, Blandine de Lauzon-Guillain, Sabine Plancoulaine, Carla Lança, Seang Mei Saw, Keith M. Godfrey, Lynette P. Shek, Kok Hian Tan, Marie-Aline Charles, Yap Seng Chong, Barbara Heude, Johan G. Eriksson, Falk Müller-Riemenschneider, Sandrine Lioret, Mary F.-F. Chong, Jonathan Y. Bernard

**Affiliations:** 1 Saw Swee Hock School of Public Health, National University of Singapore and National University Health System, Singapore, Singapore; 2 Université de Paris, Centre for Research in Epidemiology and Statistics (CRESS), Inserm, INRAE, F-75004 Paris, France; 3 Singapore Institute for Clinical Sciences (SICS), Agency for Science, Technology and Research (A*STAR), Singapore, Singapore; 4 Department of Obstetrics and Gynaecology and Human Potential Translational Research Programme, Yong Loo Lin School of Medicine, National University of Singapore, Singapore, Singapore; 5 Singapore Eye Research Institute, Singapore National Eye Centre, Singapore, Singapore; 6 Duke-NUS Medical School, Singapore, Singapore; 7 MRC Lifecourse Epidemiology Centre and NIHR Southampton Biomedical Research Centre, University of Southampton and University Hospital Southampton NHS Foundation Trust, Southampton, UK; 8 Department of Paediatrics, Yong Loo Lin School of Medicine, National University of Singapore, Singapore, Singapore; 9 Department of Maternal Fetal Medicine, KK Women’s and Children’s Hospital, Singapore, Singapore; 10 Department of General Practice and Primary Health Care, University of Helsinki and Helsinki University Hospital, Helsinki, Finland; 11 Folkhälsan Research Centre, Helsinki, Finland

**Keywords:** Preschool children, Lifestyle patterns, Diet, Physical activity, Screen time, Family ecological model, Hierarchical analysis

## Abstract

**Background:**

Integrated patterns of energy balance-related behaviours of preschool children in Asia are sparse, with few comparative analyses.

**Purpose:**

Using cohorts in Singapore (GUSTO) and France (EDEN), we characterized lifestyle patterns of children and investigated their associations with family-focused contextual factors.

**Methods:**

Ten behavioural variables related to child’s diet, walking, outdoor play and screen time were ascertained by parental questionnaires at age 5–6 years. Using principal component analysis, sex-specific lifestyle patterns were derived independently for 630 GUSTO and 989 EDEN children. Contextual variables were organised into distal (family socio-economics, demographics), intermediate (parental health, lifestyle habits) and proximal (parent-child interaction factors) levels of influence and analysed with hierarchical linear regression.

**Results:**

Three broadly similar lifestyle patterns were identified in both cohorts: “discretionary consumption and high screen time”, “fruit, vegetables, and low screen time” and “high outdoor playtime and walking”. The latter two patterns showed small differences between cohorts and sexes. The “discretionary consumption and high screen time” pattern was consistently similar in both cohorts; distal associated factors were lower maternal education (EDEN boys), no younger siblings (GUSTO boys) and Malay/Indian ethnicity (GUSTO), while intermediate and proximal associated factors in both cohorts and sexes were poor maternal diets during pregnancy, parents allowing high child control over food intake, snacking between meals and having television on while eating.

**Conclusions:**

Three similar lifestyle patterns were observed among preschool children in Singapore and France. There were more common associated proximal factors than distal ones. Cohort specific family-focused contextual factors likely reflect differences in social and cultural settings. Findings will aid development of strategies to improve child health.

**Supplementary Information:**

The online version contains supplementary material available at 10.1186/s12966-022-01266-4.

## Introduction

Early childhood is a sensitive developmental phase for the foundation of later health [1]. A child’s development is not only shaped by genetic determinants, but also influenced by a range of intrinsically connected and acquired habits [2]. The latter include energy balance-related behaviours (EBRBs) such as diet, physical activity, and sedentary behaviours (e.g. screen time) [3]. These lifestyle behaviours, established during these formative years, can track into adulthood and influence physical and mental health [1], consequently impacting on the risk of non-communicable diseases in later life.

When EBRBs are addressed individually, their interactions are not accounted for and their influence on health outcomes may be under-estimated [4–6]. Moving beyond examining individual behaviours in isolation, more recent studies have adopted data-reduction methods to identify integrated patterns of EBRBs, also known as “lifestyle patterns”. These studies have broadly classified children’s lifestyle patterns into healthy, unhealthy and mixed (i.e. combination of healthy and unhealthy EBRBs) [5–8]. For example, mixed patterns comprising high physical activity, less healthy diets, and high sedentary behaviour, were more commonly seen in boys, where girls were less physically active, similarly sedentary, and had healthier diets [5–8]. Apart from sex differences, the lifestyle patterns of children appear to be influenced by several factors such as socio-demographics of the family as well as parental beliefs, attitudes, and actions. For example, children from lower socio-economic position families generally display suboptimal lifestyle patterns [5–7]; likewise parenting practices and behaviours such as permissive parenting style [9], excessive parental screen time [10], and irregular or unfavourable mealtime habits (e.g. eating with the television on) [10, 11] have been associated with less healthy lifestyle patterns in children.

However, most studies to date have only conducted univariable analysis or standard regression models, which do not account for the hierarchical nature and interrelationships among multilevel factors [12, 13]. For example, lower socio-economic position (a distal/upstream factor) of families, by constraining the offered opportunities, can adversely influence lifestyle patterns of their children through less optimal parenting behaviours. The latter are closer to the child experience and thus considered as a more proximal/downstream factor and likely a mediator. Adjustment for these mediating factors (e.g. by including all variables in a single model) may lead to underestimation of the effects of distal factors. To overcome this limitation and to optimize the interpretation of findings, conceptual hierarchical frameworks [13] have been proposed to guide variable selection in multivariable models and holistically examine family factors associated with lifestyle patterns in children.

Additionally, most existing lifestyle patterns studies have been conducted in Caucasian children [7] and there is a paucity of studies among Asian children. There are only two studies to date focused on Asian children, one in Hong Kong [14] and one in Japan [10], and two multi-country studies with representation from Asians and Caucasians [15, 16]. Given differences in food consumption patterns, culture, and living environment, we aimed to determine whether these differences would result in different family-focused contextual factors being associated with similar lifestyle patterns among Asian and Caucasian children.

Using data from two independent mother-offspring cohorts in Singapore and France, we aimed to (i) characterize sex-specific lifestyle patterns in 5-year-olds and (ii) investigate their associations with family-focused contextual factors. We hypothesized that broadly similar lifestyle patterns would emerge from both cohorts and despite cultural and environmental differences between Singapore and France, some contextual factors would be similar.

## Methods

### Study population

We used data from the Growing Up in Singapore Towards healthy Outcomes (GUSTO) study [17] and the “Étude des Déterminants pré- et post-natals précoces de la santé de l’Enfant” (EDEN) mother-child cohort [18].

The GUSTO cohort recruited 1450 pregnant women (< 14 weeks’ gestation) from two major public maternity hospitals in Singapore from June 2009 to September 2010 (61.3% response rate). Inclusion criteria were: age 18 years and above; Singapore citizens or permanent residents; willingness to donate cord, cord blood, and placenta; intention to deliver in the study hospitals and reside in Singapore for the next 5 years; and fetus with both sets of grandparents of the same ethnicity. Women on chemotherapy or with serious health conditions, such as Type I diabetes and psychosis, were excluded. The study was approved by the National Health Care Group Domain Specific Review Board (reference D/09/021) and the Sing Health Centralized Institutional Review Board (reference 2009/280/D).

The EDEN cohort recruited 2002 pregnant women (< 24 weeks’ gestation) from two university maternity clinics in Nancy and Poitiers, France, between 2003 and 2006 (52% response rate). Exclusion criteria were history of diabetes, twin pregnancies, intention to move out of the study region within the next 3 years, and inability to speak French. The study was approved by the Ethical Research Committee (Comite consultatif de protection des personnes dans la recherche biomedicale) of Bicêtre Hospital and by the Data Protection Authority (Commission Nationale de l’Informatique et des Libertés). Informed written consent was obtained from all participants in both cohorts.

### EBRB variables

We considered 10 continuous variables related to the child’s diet, walking, outdoor play, and screen time. Data were collected using cohort-specific questionnaires administered to the parents/caregiver (mostly mothers) when children were 5 to 6 years of age (Supplemental Table 1).

#### Dietary intake

For GUSTO, the frequency of food items consumed by children at age 5 years was assessed using a validated 112-item food frequency questionnaire (FFQ) and administered by trained interviewers to parents/caregivers [19]. Caregivers had to indicate the frequency of consumption over the past month as ‘never’, ‘number of times per month’, ‘number of times per week’ or ‘number of times per day’.

For EDEN, a FFQ was completed by parents to assess children’s dietary intake at age 5–6 years [20]. In brief, this is a short version of a validated FFQ [21] that was previously used to ascertain diet among French adolescents and adults, including mothers during their pregnancy. This short FFQ included 27 food groups along with seven possible responses, ranging from “never” to “several times per day” over a usual week.

Data were harmonized across both cohorts and seven broadly similar food and drink groups were created for inclusion in the current lifestyle pattern analysis, i.e., fruit, vegetables, sugar sweetened beverages (SSBs), desserts and sweet snacks, savoury snacks, French fries, and processed meat (all measured in daily frequencies).

#### Movement behaviours

For GUSTO, time spent on walking (open response) on most recent weekday, Saturday, and Sunday was assessed using a validated preschool-age physical activity questionnaire administered at age 5.5 years [22], while time spent on outdoor play and screen (i.e.*,* television, computers, and hand-held devices) on weekdays and weekend days were collected at age 6 years in 5-min increments [23].

For EDEN, time spent on walking, outdoor play, and screen time (i.e.*,* television and playing computer games) on a typical school and non-school day (i.e.*,* Wednesday and weekend days) at age 5–6 years were reported by parents in a questionnaire [11].

In both cohorts, durations (in hours/day) were weighted according to the type of day (e.g. weekday, weekend day). Outliers were replaced by maximum values of the acceptable distributions, i.e., in GUSTO, by 5 h, 6 h, and 8 h per day for walking (*n* = 8), outdoor play (*n* = 8), and screen time (*n* = 17) respectively; and in EDEN, by 3.7 h and 5 h per day for walking (*n* = 1) and screen time (*n* = 4) respectively. Outdoor play was further standardized by season to account for seasonal variations in EDEN [11].

### Contextual variables

**Fig. 1:**
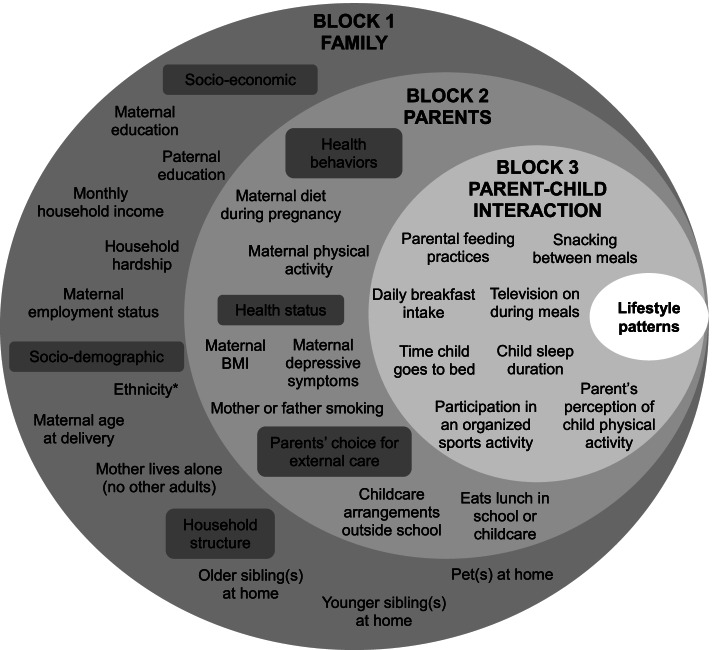
Conceptual hierarchical framework of factors influencing children’s lifestyle patterns for the GUSTO-EDEN comparative study. ^*^only available in GUSTO

The selection of the contextual variables was guided by the Family Ecological Model [24] and subject to the availability and comparability of the variables in both cohorts. The 25 chosen variables were then categorized into a three-level hierarchical framework [13], structured from distal variables (family socio-economics and demographics) (shown in the outer circle of Fig. 1) to the intermediate (parental health and lifestyle habits) and proximal (parent-child interaction factors). Of note, ethnicity was available in GUSTO only (58% Chinese, 25% Malay and 17% Indian). A detailed description of the variables is presented in Supplemental Table 2. In brief, data used in the study were either obtained from clinical records or collected by questionnaires administered during pregnancy and at different stages of the follow up— age 4, 4.5, 5, 5.5, and 6 years for GUSTO and age 2 and 5–6 years for EDEN.

### Statistical analysis

The behavioural variables were summarized by sex and by cohort; and Mann Whitney tests used to compare medians. Sex-specific lifestyle patterns were derived independently for GUSTO and EDEN children using principal component analysis (PCA) with varimax rotation on the 10 standardized behavioural variables. Patterns were retained based on eigenvalues > 1.0, scree plot examination, and the pattern interpretability [25, 26]. The lifestyle pattern score for each child was calculated by summing the standardized values of each behavioural variable weighted by the variable PCA loading. These loadings (multiplied by 100 for easier interpretation) represent correlation coefficients between the behavioural variables and the derived pattern; hence, higher scores indicate greater adherence to the derived lifestyle pattern. We characterized each pattern by variables that had absolute PCA loadings > 25 for both GUSTO and EDEN.

Univariable analyses were conducted between each contextual variable and lifestyle patterns. Variables with *P* < 0.20 in these univariable analyses were included in the final three-stage hierarchical regression analyses following Victora et al. approach [13]. In brief, Model 1 included distal variables (family socio-economics and demographics). Model 2 further included intermediate variables (parental health and lifestyle habits). Model 3 further included proximal variables (parent-child interaction factors). In the multivariable analysis, each variable of a given block was interpreted within the first model in which it was included, regardless of its performance in the subsequent model(s) [13].

For both cohorts, missing values for contextual variables were handled using the Markov chain Monte Carlo method, assuming missingness at random. The imputation models included the behavioural variables and all exposures (contextual variables). We imputed 20 datasets, and the estimates and standard errors were combined using Rubin’s rules [27]. To obtain an overall *p*-value for categorical variables, we used the median p-value rule, that consists in retaining the median p-value of the Wald tests conducted across the imputed datasets [28]. To evaluate whether the imputation of missing data may have affected the results, we carried out sensitivity analyses in participants with complete data (*n* = 296 in GUSTO and *n* = 619 in EDEN).

The significance level was set at 5%. All statistical analyses were done using Stata 14 (StataCorp LP, USA) in GUSTO and SAS®, Version 9.4 (SAS Institute) in EDEN.

## Results

### Characteristics of study population

**Fig. 2:**
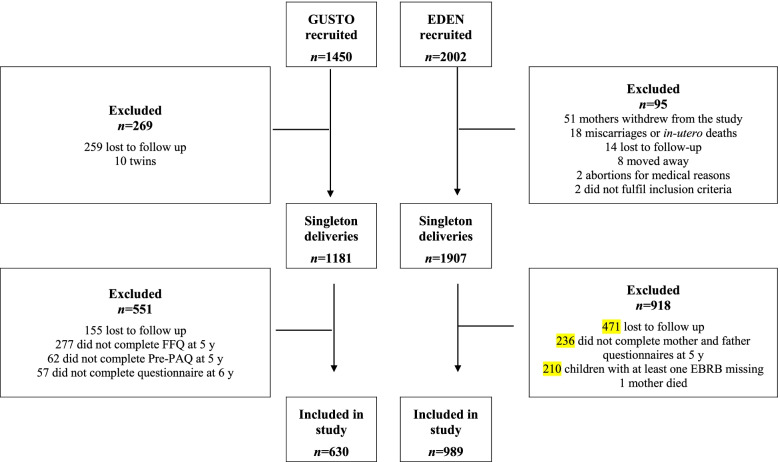
Flowchart of participants included in the analysis. FFQ: Food frequency questionnaire; Pre-PAQ: Preschool-age physical activity questionnaire

Of the singleton deliveries (GUSTO *n* = 1181; EDEN *n* = 1907), we excluded children who were lost to follow up (GUSTO *n* = 155; EDEN *n* = 471) or had incomplete questionnaires for the variables of interest, resulting in two analytic samples of 630 GUSTO and 989 EDEN children (Fig. 2**)**. Children excluded from the analysis did not differ by sex or prematurity, however, they were more likely to have a lower birth weight and born to younger mothers (Supplemental Table 3). In addition, those who were excluded from the analysis in EDEN were more likely born to less educated and multiparous mothers.

Across both cohorts, boys had higher screen time than girls [GUSTO: 2.73 (2.71) h/day vs. 2.25 (2.28) h/day; EDEN: Median (IQR) 1.29 (1.14) h/day vs. 1.14 (1.07) h/day]. There were no significant differences between boys and girls for the other behavioural variables in GUSTO. In EDEN, boys had higher intake of SSBs [1.00 (0.93) per day vs. 0.74 (0.88) per day] and spent more time outdoors than girls [− 0.11 (1.34) SD per day vs. -0.27 (1.13) SD per day] (Table 1).

**Table 1:** Behavioural variables distribution for boys and girls

	GUSTO *n* = 630	GUSTO boys *n* = 330	GUSTO girls *n* = 300		EDEN *n* = 989	EDEN boys *n* = 527	EDEN girls *n* = 462	
Behavioural variables	Median (IQR)	Median (IQR)	Median (IQR)	*p-value*	Median (IQR)	Median (IQR)	Median (IQR)	*p-value*
Fruit, freq/day	1.04 (1.00)	0.99 (1.00)	1.04 (1.04)	*0.41*	1.00 (1.14)	1.00 (1.14)	1.00 (0.86)	*0.48*
Vegetables, freq/day	1.29 (1.62)	1.29 (1.64)	1.26 (1.51)	*0.89*	1.00 (0.86)	1.00 (0.86)	1.00 (1.14)	*0.52*
Sugar sweetened beverages, freq/day	1.00 (1.07)	0.96 (1.04)	0.93 (1.08)	*0.85*	0.86 (0.98)	1.00 (0.93)	0.74 (0.88)	*< 0.001*
Desserts & sweet snacks, freq/day	1.14 (1.22)	1.11 (1.12)	1.04 (1.17)	*0.81*	1.36 (1.14)	1.36 (1.16)	1.36 (1.14)	*0.28*
Savoury snacks, freq/day	0.07 (0.14)	0.07 (0.14)	0.07 (0.14)	*0.74*	0.07 (0.05)	0.07 (0.27)	0.07 (0.05)	*0.86*
French fries, freq/day	0.09 (0.10)	0.09 (0.10)	0.07 (0.10)	*0.46*	0.07 (0.21)	0.07 (0.21)	0.07 (0.21)	*0.10*
Processed meat, freq/day	0.32 (0.43)	0.29 (0.43)	0.32 (0.43)	*0.92*	0.07 (0.21)	0.07 (0.21)	0.07 (0.21)	*0.07*
Walking, h/day	0.51 (0.88)	0.55 (0.86)	0.49 (0.86)	*0.27*	0.64 (0.52)	0.64 (0.55)	0.62 (0.52)	*0.47*
Outdoor play^a^	1.29 (1.50)	1.29 (1.35)	1.29 (1.52)	*0.53*	−0.19 (1.25)	− 0.11 (1.34)	− 0.27 (1.13)	*0.001*
Screen time, h/day	2.57 (2.71)	2.73 (2.71)	2.25 (2.28)	*0.02*	1.14 (1.00)	1.29 (1.14)	1.14 (1.07)	*0.008*

Values are median (IQR). Mann Whitney tests were used to compare medians between girls and boys

^a^Outdoor play is standardized by season in EDEN and expressed as h/day in GUSTO

### Characteristics of lifestyle patterns

**Fig. 3:**
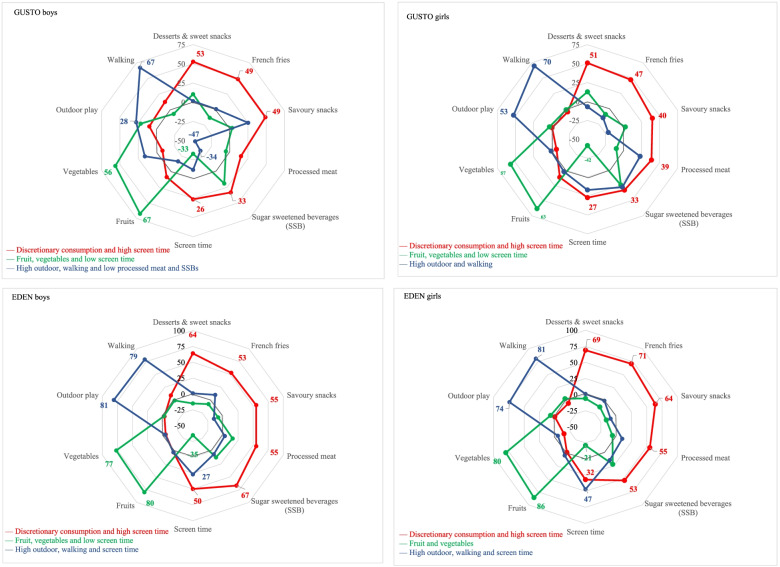
PCA loadings of lifestyle patterns

Three broadly similar lifestyle patterns were identified among the boys and girls in the EDEN and GUSTO cohorts (Fig. 3). Each of these patterns accounted for 12–23% of the explained variance.

The first pattern was positively correlated with intake of SSBs, desserts and sweet snacks, savoury snacks, fries, processed meat, and screen time. We labelled this pattern “Discretionary consumption and high screen time”.

The second pattern, “Fruit, vegetables, and low screen time”, was characterized by high intakes of fruit and vegetables, and low levels of screen time. For EDEN girls, the PCA loading for screen time was negative but below the defined threshold, and thus, it was termed “Fruit and vegetables”.

The third pattern was positively correlated with screen time, outdoor playtime, and walking in EDEN children, but only with outdoor playtime and walking in GUSTO children. We labelled this pattern “High outdoor, walking and screen time” in EDEN children and “High outdoor and walking” in GUSTO girls. For GUSTO boys, this pattern was also negatively correlated with processed meat and SSBs intake, thus, it was labelled “High outdoor, walking and low processed meat and SSBs”.

### Associations with contextual factors

Since the “Discretionary consumption and high screen time” pattern was consistent across both cohorts and sexes, we show its findings in Table 2**,** while findings from the other lifestyle patterns are presented in Supplemental Table 4.

**Table 2:** Hierarchical regression analyses to study contextual factors associated with “Discretionary foods and high screen time*”* pattern scores in GUSTO and EDEN children

	GUSTO boys *n* = 330	GUSTO girls *n* = 300	EDEN boys *n* = 527	EDEN girls *n* = 462
**Family socio-economics and demographics**				
**Maternal education**				
Low ***v.*** High	NIR	0.18 (− 0.41, 0.76)	0.35 (0.07, 0.63)*	0.30 (− 0.01, 0.62)
Intermediate ***v.*** High		0.26 (− 0.21, 0.73)	0.00 (− 0.21, 0.21)	0.05 (− 0.19, 0.28)
**Paternal education**				
Low ***v.*** High	0.19 (− 0.32, 0.70)	0.17 (− 0.44, 0.77)	0.21 (− 0.05, 0.48)	0.23 (− 0.06, 0.53)
Intermediate ***v.*** High	0.16 (− 0.28, 0.60)	− 0.12 (− 0.61, 0.36)	0.02 (− 0.21, 0.25)	0.09 (− 0.17, 0.35)
**Monthly household income**^a^				
Low ***v.*** High	− 0.08 (− 0.59, 0.42)	0.03 (− 0.55, 0.61)	0.22 (− 0.06, 0.49)	− 0.04 (− 0.36, 0.28)
Intermediate v***.*** High	0.00 (− 0.45, 0.45)	− 0.21 (− 0.73, 0.31)	0.17 (− 0.05, 0.38)	0.02 (− 0.20, 0.25)
**Household hardship**^a^				
Yes ***v.*** No	NIR	NIR	NIR	NIR
**Ethnicity**				
Malay ***v***. Chinese	1.10 (0.68, 1.52)*	1.08 (0.63, 1.53)*	NIR	NIR
Indian ***v.*** Chinese	0.47 (0.04, 0.90)*	0.61 (0.16, 1.06)*		
**Maternal age at delivery**				
< 27 y ***v***. > 33 y	0.10 (−0.34, 0.55)	0.19 (− 0.27, 0.65)	0.09 (− 0.15, 0.32)	NIR
27–33 y ***v.*** > 33 y	0.13 (− 0.24, 0.49)	− 0.01 (− 0.38, 0.36)	−0.12 (− 0.31, 0.08)	
**Maternal employment status**^a^				
Unemployed ***v***. Full-time	NIR	− 0.23 (− 0.62, 0.16)	0.08 (− 0.17, 0.32)	0.20 (− 0.07, 0.48)
Part-time ***v.*** Full-time		− 0.61 (−1.16, − 0.06)	−0.01 (− 0.20, 0.18)	0.08 (− 0.13, 0.29)
**Mother lives alone (no other adults)**^a^				
Yes ***v.*** No	NIR	NIR	NIR	NIR
**Older sibling(s) at home**				
Yes ***v.*** No	0.03 (−0.30, 0.37)	0.24 (− 0.10, 0.59)	NIR	NIR
**Younger sibling(s) at home**^a^				
Yes ***v.*** No	−0.39 (− 0.74, − 0.04)*	NIR	NIR	NIR
**Pets at home**^1^				
Dog(s) ***v.*** No pets	−0.44 (−1.31, 0.43)	NIR	0.22 (0.01, 0.42)*	0.25 (0.02, 0.48)*
Other animal(s) ***v.*** No pets	0.34 (−0.11, 0.78)		0.17 (−0.03, 0.37)	0.08 (−0.13, 0.29)
**Parental health and lifestyle habits**				
**Maternal diet during pregnancy**				
Healthy	−0.11 (− 0.29, 0.06)	−0.20 (− 0.37, − 0.03)*	0.12 (0.03, 0.22)*	NIR
Western	NIR	NIR	0.37 (0.28, 0.46)*	0.31 (0.17, 0.45)*
**Maternal physical activity**^a^				
Low ***v.*** High	NIR	0.28 (−0.15, 0.71) -0.06 (− 0.47, 0.35)	0.16 (− 0.06, 0.39) 0.09 (− 0.15, 0.33)	0.13 (− 0.15, 0.41) 0.00 (− 0.29, 0.30)
Intermediate ***v.*** High				
**Maternal BMI**^a^				
Obese ***v***. Normal	−0.01 (− 0.44, 0.42) 0.23 (− 0.13, 0.59)	0.08 (− 0.37, 0.54)	NIR	0.17 (− 0.09, 0.43)
Overweight ***v.*** Normal		0.22 (− 0.16, 0.59)		0.04 (− 0.18, 0.26)
**Maternal depressive symptoms**^a^				
Yes ***v.*** No	0.39 (−0.16, 0.93)	NIR	0.00 (−0.22, 0.22)	0.14 (−0.10, 0.38)
**Mother or father smoking**^a^				
Yes ***v.*** No	−0.04 (− 0.40, 0.32)	0.10 (− 0.29, 0.50)	0.09 (− 0.08, 0.26)	0.01 (− 0.18, 0.20)
**Childcare arrangements outside school**^a^				
Centre-based ***v.*** Parental care	0.13 (− 0.23, 0.50)	−0.41 (−1.10, 0.27)	−0.11 (− 0.29, 0.07)	−0.10 (− 0.32, 0.11)
Non-parental ***v.*** Parental care	0.56 (0.10, 1.02)*	−0.09 (− 0.63, 0.44)	−0.17 (− 0.39, 0.04)	0.08 (− 0.15, 0.30)
**Eats lunch in school or childcare**^a^				
Yes ***v.*** No	NIR	−0.12 (− 0.71, 0.47)	NIR	NIR
**Parent-child interaction factors**				
**Parental feeding practices**^b^				
High ***v.*** mid and low tertile				
Child control	0.51 (0.12, 0.91)*	0.51 (0.08, 0.93)*	0.12 (−0.05, 0.29)	0.21 (0.02, 0.40)*
Food as reward	0.13 (−0.29, 0.55)	0.42 (0.02, 0.81)*	0.02 (−0.14, 0.19)	0.01 (−0.17, 0.19)
Restriction for health	NIR	NIR	−0.09 (− 0.25, 0.07)	−0.14 (− 0.31, 0.03)
Pressure to eat	NIR	NIR	NIR	NIR
**Daily breakfast intake**^a^				
Yes ***v.*** No	NIR	−0.37 (−0.81, 0.07)	NIR	NIR
**Television on during meals**^a^				
Often ***v.*** Never	0.19 (−0.18, 0.56)	0.58 (0.20, 0.96)*	0.52 (0.31, 0.72)*	0.35 (0.12, 0.58)*
Sometimes ***v.*** Never	0.31 (−0.04, 0.66)	0.35 (−0.02, 0.72)	0.38 (0.20, 0.56)*	0.29 (0.09, 0.49)*
**Snacking between meals**^a^				
Often ***v.*** Never	1.01 (0.52, 1.49)*	0.83 (0.34, 1.32)*	0.66 (0.41, 0.91)*	0.85 (0.58, 1.13)*
Sometimes ***v.*** Never	0.52 (0.15, 0.90)*	0.21 (−0.18, 0.61)	0.07 (−0.11, 0.25)	0.06 (− 0.13, 0.26)
**Participation in an organized sports activity**^a^				
Yes ***v.*** No	−0.15 (− 0.46, 0.16)	−0.15 (− 0.47, 0.17)	−0.15 (− 0.30, 0.00)*	−0.06(− 0.23, 0.11)
**Parent’s perception of child’s physical activity**^a^				
More active ***v.*** Less or as active than other children	NIR	NIR	NIR	−0.08 (− 0.34, 0.18)
**Time child goes to bed**^a^	0.17 (−0.09, 0.43)	0.06 (− 0.17, 0.29)	0.06 (− 0.15, 0.27)	0.19 (− 0.04, 0.42)
**Child’s sleep duration**^a^	NIR	NIR	−0.12 (− 0.31, 0.06)	−0.05 (− 0.26, 0.16)

^a^When the child was between age 4 to 6 years in GUSTO and age 5 years in EDEN

^b^When the child was age 5 years in GUSTO and age 2 years in EDEN

*denote *P* < 0.05

*NIR* Not Included in final hierarchical regression. Variables with *p > 0.20* in the univariate analyses and also in the multivariable analysis of the n-1 block were not included in the final three-stage hierarchical regression analyses

Distal factors found to be associated with the “Discretionary consumption and high screen time” pattern differed between the two cohorts. Scores for this pattern were higher in EDEN boys whose mothers had lower educational level (mean difference (95% CI): 0.35 [0.07, 0.63]) between low and high maternal education levels). In GUSTO, adherence to this pattern was greater in Malay (1.10 [0.68, 1.52] in boys; 1.08 [0.63, 1.53] in girls) and Indian (0.47 [0.04, 0.90] in boys; 0.61 [0.16, 1.06] in girls) than in Chinese children. GUSTO boys with no younger siblings (i.e. the youngest child or only child) also scored higher than boys with younger siblings (0.39 [0.04, 0.74]).

For the intermediate level of contextual factors, less healthy maternal diets during pregnancy were associated with higher scores on this lifestyle pattern across both cohorts and sexes. In addition, scores for this pattern were higher in GUSTO boys in partial centre-based childcare and who had non-parents as primary caregivers. Other variables from the intermediate level were not related to this lifestyle pattern.

Of the proximal factors and across both cohorts and sexes, we observed higher scores in children who often had television on while eating and snacked between meals. Parents were also more likely to allow high child control over food intake when children scored higher in this lifestyle pattern. In addition, scores for this pattern were higher in EDEN boys who did not participate in an organized physical activity and higher scores in GUSTO girls were associated with parents using food as reward. Sleep variables were not related to scores on this pattern.

Across both cohorts, lower scores on “Fruit, vegetables, and low screen time” were associated with mothers/fathers with lower educational level (distal factor) and less healthy maternal diets during pregnancy (intermediate factor) (Supplemental Table 4). Lower adherence was also observed in children who often had television on while eating (proximal). There were no common contextual factors associated with the third pattern across both cohorts.

Results from sensitivity analyses showed largely similar findings and direction of point estimates were the same (data not shown). Imputation of missing data did not alter our conclusion.

## Discussion

In these two different population and cultural environments (Singapore versus France), we identified three broadly similar lifestyle patterns among 5–6-year-old children. A “discretionary consumption and high screen time” pattern was highly similar across boys and girls in both GUSTO and EDEN cohorts. Compared to distal and intermediate factors, there were more common proximal factors associated with this pattern across both cohorts and sexes: poor maternal diets during pregnancy, parents allowing high child control over food intake, snacking between meals and having television on while eating.

Similar to the “discretionary consumption and high screen time” pattern observed in our study, the co-occurrence of unhealthy snacks and SSBs consumption and screen-based sedentary behaviours have been noted by various studies among children of different regions and age groups [5–8], starting as early as 18 months [29] and 2 years of age [30]. Our findings add evidence to the combinations of EBRBs despite diverse cultures across different countries [31]. Although the cross-sectional nature of the design does not allow any causal inference, some assumptions may be drawn from the current findings. The common contextual factors identified in both cohorts support the potential mechanisms underlying this pattern. Our results suggest that poor diets during pregnancy, which often continues after pregnancy [32], and parents allowing high child control over food intake (akin to permissive parenting styles) [9] were strongly associated with combination of unhealthy behaviours in young children as they are mostly influenced by their parents, who are usually their main role models and providers. In addition, children who adhere to this pattern often had television on while eating, which promotes passive snacking and potentially overconsumption due to distraction from satiety cues [33]. There is also a possibility that exposure to unhealthy food advertisements when watching television increases children’s preferences for nutritionally-poor foods and higher snacking frequency [34]. Taken together, our findings highlight the importance of providing guidance on parenting in shaping healthy child behaviours and these proximal factors are potential entry points of interventions.

Focusing on the distal factors, findings differed between GUSTO and EDEN cohorts. In EDEN, lower maternal education was associated with higher adherence to the “discretionary consumption and high screen time” pattern in children. Consistent with previous findings [5–7], parents’ poor health literacy and knowledge may be associated with suboptimal child’s dietary intake and screen-based behaviours. In GUSTO, scores for this pattern were higher in children of Malay and Indian ethnicity compared to those of Chinese ethnicity, but no associations were found with maternal education. This finding is supported by a study among Singapore families which demonstrated that the influence of ethnicity and culture on parenting styles is greater than parental education [35]. In addition, other studies in Singapore have also reported that Malay children have lower diet quality scores compared to other ethnic groups [36, 37]. It is hypothesized that there may be limitations in culturally acceptable or available prudent food options for Malay families and this warrant further consideration in designing future interventions [36].

There were other cohort-specific contextual factors of the “discretionary consumption and high screen time” pattern worth noting. In EDEN, the lack of participation in organized physical activity was associated with higher adherence to the “Discretionary consumption and high screen time” pattern. However, this was only observed in boys, but not in girls. This sex-specific association, consistent with previous research in adolescents [38], could be attributed to the high screen time usage in boys compared to girls, likely to displace the time for other physical activities [38]. In GUSTO boys, being the youngest child (or only child) and having non-parents (i.e. grandparents or domestic helpers) as primary caregivers were associated with unhealthy lifestyle patterns. This may be due to overindulgence and/or poor awareness of health recommendations among domestic helpers and grandparents [39, 40]. Overindulgence might underlie the observed association with younger siblings, as Alder’s birth order theory suggests that the youngest child is more pampered with lack of independence [41]. In GUSTO girls, the practice of using food as reward is related to unhealthy eating habits [42]. The hypothesis or mechanisms related to cohort-specific or sex-specific findings are unclear as there are limited studies comparing Asians and Europeans; and even fewer have examined sex differences. However, our findings might provide valuable population specific information on prioritizing areas for action. Interventions involving multiple levels of influence such as enhancing health literacy, improving parenting practices, along with greater accessibility to prudent food options may be effective to improve the health and well-being of our children.

The second pattern, “Fruit, vegetables, and low screen time”, has been less frequently identified in previous studies [5–7]. Fruit and vegetables were usually reported with the co-occurrence of physical activity behaviours but this pattern was not observed in our study. As the contextual factors associated with the second pattern were similar to the first pattern (i.e. maternal diets and having television on during meals), these are relevant levers to focus on when designing future interventions, as they are likely to simultaneously and favourably impact both unhealthy and healthy combinations of EBRBs.

In this study, high physical activity level (as approximated by outdoor play and walking) was observed co-existing with high screen-based sedentary behaviours in EDEN children. Although a mixed physical activity and sedentary behaviour pattern is prevalent in the literature, this is the first study, to our knowledge, to identify a “high outdoor, walking and screen time” pattern in children at this young age group. Anecdotal evidence suggest that it is generally safe for children to walk and play outdoors in the studied cities. Of note, the term ‘high outdoor’ is relative to our population activity level and does not necessarily reflect a higher amount of outdoor time when compared with other studies. The combination of high physical activity and high screen time may be explained by the competitive element in both sports and computer games, which appeal to certain children [43]. It is also hypothesized that children may be active during the day or after school and then watch television or engage in other screen-based activities in the evenings [44]. Further studies are needed to understand the co-occurrence of high screen time and high level of physical activities in this age group.

Previous studies, which were mostly conducted in older children or adolescents, have identified sex differences in the clustering of lifestyle behaviours [5–7] but this was less apparent in our study of younger children. One possible explanation is that the lifestyle and movement behaviours of children become increasingly different as they grow older [45]. For example, physical activity levels in girls were similar to boys during childhood, but decline dramatically during adolescence due to their advanced pubertal maturation compared with boys [46].

Several limitations of our study should be noted. First, although detailed frequency and duration of each behaviour was collected from questionnaires, data were based on self-reports by parents which may misestimate the child’s health behaviours and suffer recall bias. Studies have shown moderate positive correlations between parent-report and direct measures of physical activity in preschool children but weaker correlation for screen time [47–50]. For diet, the FFQs used in this study have been validated and have shown reasonable agreement with dietary records [19, 21]. Second, there were differences in questionnaire administration between both cohorts. For example, children reached 5 years of age earlier in EDEN (2010–2012) than in GUSTO (2014–2015), and in the meantime handheld screens have emerged. The media environment has changed, with the incursion of new technological devices (smartphones or tablets), when the study was conducted in Singapore which may have led to differences in screen behaviours findings between the two cohorts. There were also differences in the phrasing of questions between both cohorts but we managed to align and match the variables and categories as much as possible to minimize the differences. Third, we did not include sleep duration as part of the lifestyle patterns as it would have reduced our sample size significantly due to missing data. Instead, we used multiply-imputed sleep variables as a contextual factor so that such a valuable dimension was not completely left out. Fourth, children excluded from the analyses tended to have lower birth weights and born to younger mothers. In addition, those who were excluded from the analysis in EDEN were more likely born to multiparous and less educated mothers (a proxy for socio-economic position). These characteristics may have implications for generalizability of our study. In particular, children from lower socio-economic position families generally display suboptimal lifestyle patterns [4–6] and our effect estimates could have been underestimated, but this should be further investigated. Last, although we considered many contextual factors, our findings could still be influenced by residual and unmeasured factors (e.g. built environment and community-related correlates) and causality cannot be claimed, as in any single observational study.

Strengths of our study include the analysis of preschool children from two geographically and culturally diverse countries, the comprehensive assessment of a wide range of contextual factors, the integrative study of health behaviours, and the use of multilevel models that took into account the hierarchical relationships among the contextual factors.

In conclusion, three broadly similar lifestyle patterns were observed among preschool children in Singapore and France: unhealthy, healthy and mixed. The unhealthy “discretionary consumption and high screen time” pattern was highly similar across boys and girls in both cohorts. There were more common proximal factors across both cohorts and sexes than distal and intermediate factors, which highlight the importance of providing guidance to parents in shaping healthy behaviours as early as possible in childhood. Contextual factors unique to specific cohorts could be attributed in part to the differences in social and cultural settings. Findings will provide valuable information to each population on prioritizing areas for action and aid in intervention development and prevention strategies to improve the health and well-being of our children from their early years.

## Supplementary Information

**Additional file 1.**

**Additional file 2.**

**Additional file 3.**

**Additional file 4.**

## Data Availability

Data described in the manuscript and analytic code will be made available upon request pending approval from our study executives. Please contact the corresponding author for more information (sandrine.lioret@inserm.fr).

## Abbreviations

EBRBs: Energy balance-related behaviours
EDEN: Étude des Déterminants pré- et post-natals précoces de la santé de l’Enfant
FFQ: Food frequency questionnaire
GUSTO: Growing Up in Singapore Towards healthy Outcomes
PCA: Principal component analysis
SSBs: Sugar sweetened beverages
