# Supplementary material for: Family-focused contextual factors associated with lifestyle patterns in young children from two mother-offspring cohorts: GUSTO and EDEN
Source: Int J Behav Nutr Phys Act. 2022 Mar 15;19:26. doi: 10.1186/s12966-022-01266-4 (PMC8922741; doi:10.1186/s12966-022-01266-4)
Supplement: Supplementary file 1 — Additional file 1. [file 12966_2022_1266_MOESM1_ESM.docx]

## Supplemental Table 1. Description of the items composing the harmonized behavioural variables

| **Behavioural variables** | **EDEN** | **GUSTO** |
| --- | --- | --- |
| **Dietary variables** | Questionnaire completed by parents^1^ when the child was age 5 years | A food frequency questionnaire was administered to mothers by interviewers when the child was age 5 years |
| **Vegetables** | Cooked vegetables + Raw vegetables | Potatoes + Sweet potatoes + Carrot, pumpkins + Fresh peas and beans + Corn + Tomato, red/green peppers + Dark green leafy vegetables + Pale coloured vegetables + Broccoli, cauliflower + Stalk vegetables + Gourds |
| **Fruit** | Fresh fruits + Stewed fruits | Apples and pears + Bananas + Papaya, + Oranges or citrus fruits + Grapes and berries, + Stone fruits + Tropical fruits + Watermelon + Dragon fruit + Guava + Kiwifruit + Canned fruits |
| **Processed meat** | Processed meat | Commercially prepared meatball + Dried, preserved meat + Processed fish and seafood products + Chicken/fish in batter/breadcrumbs |
| **French Fries** | French Fries | French Fries |
| **Sugar sweetened beverages** | Fruit juice + Carbonated soft drinks | Milo + Horlicks + Cultured drink + Yogurt drink + Milk shake + Lassi + Pure fruit Juice + Sweetened drinks + Soya Milk + Traditional drinks + Coffee/Tea with sugar |
| **Sweet snacks** | Biscuits + Chocolate | Sponge cakes/steamed cakes + Butter/cream cakes + Puff pastries + Plain crackers/rice crackers + Biscuits/cookies + Chocolate + Sweets + Jellies, pudding + Ice-cream + Dessert in soup |
| **Savoury snacks** | Crisps | Fried snacks *(e.g. chips, prawn crackers, donuts, you tiao)* |
| **Walking** | When the child was age 5 years, parents were asked how much time their child spent on walking or commuting (e.g. to school, to the nanny, to go shopping with you) on weekdays, Wednesday, and on a weekend day.  Methodology: The average walking duration (in hours) per day was calculated as follows: [(Weekday × 4) + (Wednesday) + (Weekend × 2)] / 7 | When the child was age 5.5 years, mothers were asked how much time their child spent on the following activities on weekdays, Saturday, and Sunday:   - Walked at a leisurely or moderate pace (for any reason – not just when going on a walk) - Walked at a fast pace - Walked up steep slopes   Methodology: The average activity-specific time (in hours) per day was calculated as follows: ([Weekday × 5 + Saturday + Sunday]/7). Total walking time was calculated as the sum of the above three activities. |
| **Outdoor play** | When the child was age 5 years, parents were asked how much time their child spent playing outside (e.g. in a garden, a park) outside of school and out-of-school hours cares on weekdays, Wednesday, and on a weekend day.  Methodology: The average outdoor play duration (in hours) per day was calculated as follows: [(Weekday × 4) + (Wednesday) + (Weekend × 2)] / 7 | When the child was age 6 years, mothers were asked how much time in 5-min increments, their child spent on the following activities on weekdays and weekend days in the past month:     - Playing/exercising out of doors [activities that require physical exertion] (e.g. In a backyard, walk, bike riding) - Outdoor leisure activities (Family BBQs, Park, Picnic, Beach)   Methodology: The average activity-specific time (in hours) per day was calculated as follows: ([Weekday × 5 + Weekend day × 2]/7). Total outdoor play was calculated as the sum of the above two activities |
| **Screen time** | When the child was age 5 years, parents were asked how much time their child spent on watching television, playing video or computer games on weekdays, Wednesday, and on a weekend day.  Methodology: The average screen time (in hours) per day was calculated as follows: [(Weekday × 4) + (Wednesday) + (Weekend × 2)] / 7 | When the child was age 6 years, mothers were asked how much time in 5-min increments, their child spent on the following activities on weekdays and weekend days in the past month:     - Watches television and/or play television games (e.g., PlayStation®, Wii™, Xbox™) - Uses a computer/plays computer - Plays handheld video games or uses handheld devices like handphone to play games/watch videos (e.g., Game Boy®, handphone games),   Methodology: The average device-specific time (in hours) per day was calculated as follows: ([Weekday × 5 + Weekend day × 2]/7). Total screen viewing time was calculated as the sum of the above three activities. |
