# Supplementary material for: Family-focused contextual factors associated with lifestyle patterns in young children from two mother-offspring cohorts: GUSTO and EDEN
Source: Int J Behav Nutr Phys Act. 2022 Mar 15;19:26. doi: 10.1186/s12966-022-01266-4 (PMC8922741; doi:10.1186/s12966-022-01266-4)
Supplement: Supplementary file 2 — Additional file 2. [file 12966_2022_1266_MOESM2_ESM.docx]

## Supplemental Table 2. Description of contextual variables

| **Variables** | **EDEN** | **GUSTO** |
| --- | --- | --- |
| **Family socio-economics and demographics** | | |
| **Maternal education** | Questionnaire administered to mothers by interviewers at 24–28 weeks’ gestation | Questionnaire administered to mothers by interviewers when the child was age 5 |
|  | - Low: Below high school - Intermediate: High school to 2-y university degree - High: 3-y university degree and above | - Low: Secondary and below - Intermediate: Post-secondary - High: University and above |
| **Paternal education** | Questionnaire completed by fathers at inclusion   - Low: Below high school - Intermediate: High school to 2-y university degree - High: 3-y university degree and above | Questionnaire administered to mothers by interviewers when the child was age 5   - Low: Secondary and below - Intermediate: Post-secondary - High: University and above |
| **Monthly household monthly** | Questionnaire completed by mothers when the child was age 5   - Low: < 2300 euros - Intermediate: 2301-3800 euros - High: ≥ 3801 euros | Questionnaire administered to mothers by interviewers when the child was age 5   - Low: < 4000 sgd - Intermediate: 4000-7999 sgd - High: ≥ 8000 sgd |
| **Household hardship** | Questionnaire completed by mothers when the child was age 5  Perceived financial hardship over the last year to purchase each of the following goods for the family: food, clothes, accommodation related bills, or medical care/drugs   - Yes (at least one among these four) - No | Questionnaire completed by mothers when the child was age 4  Events that have a negative impact on the family over the last year such as foreclosure on mortgage or loan, borrowing money, being worse off financially   - Yes - No |
| **Ethnicity** | There is only a small proportion (< 5%) of immigrated mothers. Due to the low variation, this variable was not included in the analysis. | Questionnaire administered to mothers by interviewers at 14 weeks’ gestation   - Chinese - Malay - Indian |
| **Maternal age at delivery** | Obtained from obstetric records | Questionnaire administered to mothers by interviewers at 14 weeks’ gestation |
|  | - <27 y - 27-33 y - >33 y | - <27 y - 27-33 y - >33 y |
| **Maternal employment status** | Questionnaire completed by mothers when the child was age 5   - Not working - Part-time - Full-time | Questionnaire administered to mothers by interviewers when the child was age 5   - Not working - Part-time - Full-time |
| **Mother lives alone (no other adults)** | Questionnaire completed by mothers when the child was age 5   - Yes - No | Questionnaire administered to mothers by interviewers when the child was age 5   - Yes - No |
| **Older sibling(s) at home** | Obtained from obstetric records   - Yes (at least one) - No | Obtained from obstetric records   - Yes - No |
| **Younger sibling(s) at home** | Obtained from questionnaire completed by mothers when the child was age 5   - Yes (at least one) - No | Questionnaire administered to mothers by interviewers when the child was age 4   - Yes - No |
| **Pet(s) at home** | Questionnaire completed by parents^1^ when the child was age 5   - No pets - At least one dog - No dog but other animal(s) | Questionnaire administered to mothers by interviewers when the child was age 5.5   - No pets - At least one dog - No dog but other animal(s) |
| **Parental health and lifestyle habits** | | |
| **Diet during pregnancy** | Maternal diet in the last trimester of pregnancy was assessed retrospectively using a validated 137-item food frequency questionnaire completed at child birth (1). | Maternal diet was assessed using a 24-h recall at 26–28 weeks’ gestation (2) |
|  | - Healthy - Western | - Healthy: Vegetable, fruit, white rice - Western: Pasta, cheese, processed meat |
| **Maternal physical activity** | Questionnaire completed by mothers when the child was age 5  Any leisure sports practiced at the time   - Low: 0 h/week - Intermediate: 0 to 120 min/week - High: >120 min/week | Questionnaire administered to mothers by interviewers when the child was age 6  Moderate and strenuous exercises during the past 6 months:   - Low: 0 h/week - Intermediate: 0-149 min/week - High: ≥150 min/week |
| **Maternal BMI** | Height was measured at 24–28 weeks’ gestation while weight was measured when the child was age 5 | Height and weight were measured when the child was age 4 |
|  | - Normal: <25 - Overweight: 25 to <30 - Obese: ≥30 | - Normal: <23 - Overweight: 23 to <27.5 - Obese: ≥ 27.5 |
| **Maternal depressive symptoms** | The Centre for Epidemiologic Studies Depression Scale (3) was self-administered by the mothers when the child was age 5 to assess depressive symptoms | The Beck Depression Inventory-II (4) was self-administered by the mothers when the child was age 4.5 to assess depressive symptoms |
|  | - Yes (cut-off: 16) - No | - Yes (cut-off:13) - No |
| **Mother or father smoking** | Questionnaires completed by mothers and fathers separately when the child was age 5 | Questionnaire administered to mothers by interviewers when the child was age 5 |
|  | - At least one of the two parents - Neither parent | - At least one of the two parents - Neither parent |
| **Childcare arrangements outside school** | Questionnaire completed by parents^1^ when the child was age 5   - Out-of-school-hours care service - Cared by mother, father, or family members - Cared by non-family members | Questionnaire administered to mothers by interviewers when the child was age 5   - Full-time centre-based childcare - Partial centre-based and parental care - Partial centre-based and non-parental care |
| **Eats lunch in school or childcare** | Questionnaire completed by parents^1^ when the child was age 5   - Yes (every day) - No | Questionnaire administered to mothers by interviewers when the child was age 5   - Yes - No |
| **Parent-child interaction factors** | | |
| **Parental feeding practices** | The Comprehensive Feeding Practices Questionnaire (5) completed by parents^1^ when the child was age 2. Divided into tertile categories of high, medium, and low scores and then dichotomized into high scores *v.* medium and low scores. | The Comprehensive Feeding Practices Questionnaire (5) was administered to the mothers when the child was age 5. Divided into tertile categories of high, medium, and low scores and then dichotomized into high scores *v.* medium and low scores. |
|  | - Child control - Food as reward - Restriction for health - Pressure to eat | - Child control - Food as reward - Restriction for health - Pressure to eat |
| **Daily breakfast intake** | Questionnaire completed by parents^1^ when the child was age 5   - Yes - No | Questionnaire completed by mothers when the child was age 4   - Yes - No |
| **Television on during meals** | Questionnaire completed by parents^1^ when the child was age 5   - Never - Sometimes - Often/always | Questionnaire completed by mothers when the child was age 5.5   - Never - Sometimes - Often/always |
| **Snacking between meals** | Questionnaire completed by parents^1^ when the child was age 5   - Never - Sometimes - Often/always | Questionnaire completed by mothers when the child was age 5   - Never - Sometimes - Often/always |
| **Participation in organized sports activity** | Questionnaire completed by parents^1^ when the child was age 5   - Yes - No   *There are 50% boys and 60% girls who participated in organized physical activity but the specific type of activity is not known.* | Questionnaire completed by mothers when the child was age 5.5   - Yes - No   *Common activities undertaken by GUSTO children were swimming, dance/physical culture, followed by gym-type program.* |
| **Parent’s perception of child’s physical activity** | Questionnaire completed by parents^1^ when the child was age 5   - More active - Less or as active than other children | Questionnaire completed by mothers when the child was age 5.5   - More active - Less or as active than other children |
| **Time child goes to bed** | Questionnaire completed by parents^1^ when the child was age 5  Averaged weekday and weekend timing | Questionnaire completed by mothers when the child was age 5.5  Averaged weekday and weekend timing |
| **Child’s sleep duration** | Questionnaire completed by parents^1^ when the child was age 5  The average sleep duration (in hours) per day was calculated as follows:  $\frac{Weekday \times5+Weekend day\times2}{7}$ | Questionnaire completed by mothers when the child was age 5.5  The average sleep duration (in hours) per day was calculated as follows:  $\frac{Weekday \times5+Weekend day\times2}{7}$ |

^1^Mostly mothers completed the questionnaires.

References

1. Yuan WL, Nicklaus S, Lioret S, Lange C, Forhan A, Heude B, et al. Early factors related to carbohydrate and fat intake at 8 and 12 months: results from the EDEN mother–child cohort. Eur J Clin Nutr. 2017;71(2):219-26.

2. Chia A-R, de Seymour JV, Colega M, Chen L-W, Chan Y-H, Aris IM, et al. A vegetable, fruit, and white rice dietary pattern during pregnancy is associated with a lower risk of preterm birth and larger birth size in a multiethnic Asian cohort: the Growing Up in Singapore Towards healthy Outcomes (GUSTO) cohort study. The American Journal of Clinical Nutrition. 2016;104(5):1416-23.

3. Radloff LS. The CES-D Scale: A Self-Report Depression Scale for Research in the General Population. Applied Psychological Measurement. 1977;1(3):385-401.

4. BECK AT, WARD CH, MENDELSON M, MOCK J, ERBAUGH J. An Inventory for Measuring Depression. Archives of General Psychiatry. 1961;4(6):561-71.

5. Musher-Eizenman D, Holub S. Comprehensive Feeding Practices Questionnaire: validation of a new measure of parental feeding practices. J Pediatr Psychol. 2007;32(8):960-72.
