# Supplementary material for: Family-focused contextual factors associated with lifestyle patterns in young children from two mother-offspring cohorts: GUSTO and EDEN
Source: Int J Behav Nutr Phys Act. 2022 Mar 15;19:26. doi: 10.1186/s12966-022-01266-4 (PMC8922741; doi:10.1186/s12966-022-01266-4)
Supplement: Supplementary file 3 — Additional file 3. [file 12966_2022_1266_MOESM3_ESM.docx]

## Supplemental Table 3. Characteristics of included and excluded participants^1^

|  | **GUSTO** | | |  | **EDEN** | | |
| --- | --- | --- | --- | --- | --- | --- | --- |
| Characteristics | Included  n^2^=630 | Excluded  n^2^=551 | *P-value* |  | Included  n^2^=989 | Excluded  n^2^=918 | *P-value* |
| Maternal age at delivery, y | 31.1 ± 5.2 | 30.1 ± 4.9 | *0.001** |  | 30.2 ± 4.7 | 28.7 ± 5.0 | *<0.001** |
| Maternal education, *n*(%) |  |  | *0.24* |  |  |  | *<0.001** |
| Low | 180 (28.6) | 171 (31.7) |  |  | 195 (20.0) | 345 (38.4) |  |
| Intermediate | 212 (33.7) | 190 (35.2) |  |  | 396 (40.5) | 350 (39.0) |  |
| High | 238 (37.8) | 179 (33.2) |  |  | 385 (39.5) | 203 (22.6) |  |
| Male, *n*(%) | 330 (52.4) | 290 (52.9) | *0.85* |  | 527 (53.3) | 473 (51.7) | *0.50* |
| Preterm birth, *n*(%) | 38 (6.0) | 49 (9.1) | *0.05* |  | 57 (5.8) | 53 (5.8) | *0.98* |
| First child, *n*(%) | 283 (44.9) | 252 (45.9) | *0.71* |  | 471 (47.8) | 376 (41.1) | *0.004** |
| Birth weight, g | 3117 ± 443 | 3040 ± 483 | *0.005** |  | 3240 ± 427 | 3173 ± 430 | *0.001** |
| ^1^Values are means ± SD or *n* (%). Chi-square and Student t tests were used to compare frequencies and means, respectively.  ^2^ n max.  *denote *P* < 0.05 | | | | | | | |
